# Yeasts and bacterial biosurfactants as demulsifiers for petroleum derivative in seawater emulsions

**DOI:** 10.1186/s13568-017-0499-6

**Published:** 2017-11-15

**Authors:** Fernanda Cristina P. Rocha e Silva, Bruno Augusto C. Roque, Nathalia Maria P. Rocha e Silva, Raquel D. Rufino, Juliana M. Luna, Valdemir A. Santos, Ibrahim M. Banat, Leonie A. Sarubbo

**Affiliations:** 1 Northeast Biotechnology Network, Federal Rural University of Pernambuco, Recife, Rua Manoel de Medeiros, s/n, Dois Irmãos, Recife, Pernambuco Zip Code: 52171-900 Brazil; 2 Advanced Institute of Technology and Innovation (IATI), Rua Joaquim de Brito, 216, Boa Vista, Recife, Pernambuco Zip Code: 50070-280 Brazil; 3 Centre for Sciences and Technology, Catholic University of Pernambuco, Rua do Príncipe, n. 526, Boa Vista, Recife, Pernambuco Zip Code: 50050-900 Brazil; 4 Faculty of Life and Health Sciences, School of Biomedical Sciences, University of Ulster, Coleraine, Northern Ireland UK; 5 Centre for Biological Sciences and Health, Catholic University of Pernambuco, Rua do Príncipe, n. 526, Boa Vista, Recife, Pernambuco Zip Code: 50050-900 Brazil

**Keywords:** *Pseudomonas*, *Bacillus*, *Candida*, Demulsification, Cell hydrophobicity, Interfacial tension, Oil, Environmental contamination

## Abstract

Oil sludge or waste generated in transport, storage or refining forms highly stable mixtures due to the presence and additives with surfactant properties and water forming complex emulsions. Thus, demulsification is necessary to separate this residual oil from the aqueous phase for oil processing and water treatment/disposal. Most used chemical demulsifiers, although effective, are environmental contaminants and do not meet the desired levels of biodegradation. We investigated the application of microbial biosurfactants as potential natural demulsifiers of petroleum derivatives in water emulsions. Biosurfactants crude extracts, produced by yeasts (*Candida guilliermondii*, *Candida lipolytica* and *Candida sphaerica*) and bacteria (*Pseudomonas aeruginosa*, *Pseudomonas cepacia* and *Bacillus* sp.) grown in industrial residues, were tested for demulsification capacity in their crude and pure forms. The best results obtained were for bacterial biosurfactants, which were able to recover about 65% of the seawater emulsified with motor oil compared to 35–40% only for yeasts products. Biosurfactants were also tested with oil-in-water (O/W) and water-in-oil (W/O) kerosene model emulsions. No relationship between interfacial tension, cell hydrophobicity and demulsification ratios was observed with all the biosurfactants tested. Microscopic illustrations of the emulsions in the presence of the biosurfactants showed the aspects of the emulsion and demulsification process. The results obtained demonstrate the potential of these agents as demulsifiers in marine environments.

## Introduction

Waste oil generated by the transport industry or from the storage and refining process forms highly stable mixtures due to the natural presence of surfactant components (asphaltenes, resins, naphthenic acids, etc.), added chemical surfactants (additives) and natural solid particles (clay and wax) in its composition (Jiang et al. 2010). Such residues are usually composed of 30–90% oil, 30–70% water and 2–15% solids by mass and are present as a complex type of water-in-oil (W/O) emulsion (Yang et al. 2005; Zhang et al. 2012). These emulsions cannot be directly disposed of in the environment due to high water content and toxicity to microorganisms and to human health (Cambiella et al. 2006). Thus, demulsification is necessary to separate this residual oil from the oil and aqueous phases, so that the recovered oil can be returned to the refining process when it contains < 0.5% water while the separated water, with reduced oil content can be discharged through the conventional wastewater treatment processes (Xia et al. 2010).

From a process point of view, oil producers are often interested in three aspects of demulsification: (1) the speed at which this separation takes place; (2) the quality of separated water for disposal; and (3) the amount of water left in the crude oil after separation. Produced oil generally has to meet company and pipeline specifications. This standard depends on company and pipeline specifications (http://petrowiki.org/Oil_demulsification#Mechanisms_involved_in_demulsification).

Crude W/O emulsions can be broken down using different methods, such as membrane separation, ionic liquids, ultra-centrifugation and electro-sedimentation (Feng et al. 2009). Among the chemical demulsifiers, anionic (naphthenic acids and fatty acids) and nonionic [polysorbate (Tween) and phenol poly oxyethylene octyl ether (PO)] surfactants (Staiss et al. 1991) emerged in the first half of the 20th century and a polyether surfactant (vinyl alkoxylated polymer) emerged later, representing the 3rd generation of chemical demulsifiers (Stephenson 1990). Despite their effectiveness, chemical demulsifiers are harmful to the environment, exerting a negative impact on marine life; such substances also do not degrade readily, which could result in the future ban of these products (Huang et al. 2009).

In comparison, natural, mainly microbial biosurfactants, are characterized by a diversified structure, excellent surface properties, reduced toxicity and environmental compatibility (Santos et al. 2016). The amphipathic characteristics of these agents allow several properties such as detergency, emulsification, demulsification, lubrication, foaming, solubilization and phase dispersion, which allows application in the recovery of water and soil contaminated by hydrocarbons (Silva et al. 2014), heavy metals (Sarubbo et al. 2015), and cleaning of oil spills (Almeida et al. 2016), as well as in other industries.

Biosurfactants are produced by microbial cultures grown on water miscible and/or immiscible substrates and are generally classified into low molecular-mass molecules (lipopeptides, glycolipids and phospholipids) and high molecular-mass polymers (polymeric and particulate surfactants). Rhamnolipids, sophorolipids and trehalolipids are the best known glycolipids, while the lipopeptide Surfactin is one of the most powerful biosurfactant (Almeida et al. 2016).

Biosurfactants reduce surface and interfacial tension, thereby increasing the solubility of hydrophilic molecules. At a given concentration of surfactant, molecular aggregations, denominated micelles are formed. The critical micelle concentration (CMC) is that in which the lowest stable surface tension is reached (Santos et al. 2016).

Emulsions are colloidal system of two immiscible liquids, wherein a liquid phase is dispersed and suspended in the form of small droplets, the dimensions of which range from 1 nm to 1 μm, in a second liquid (continuous phase). This is only possible in the presence of sufficient emulsifying agent and energy input. Depending on the liquid arrangement in the continuous phase, the emulsions are classified as water-in-oil (W/O) or oil-in-water (O/W). The most common type of oil emulsion is W/O because of the hydrophobic nature of stabilizing agents present in petroleum. Emulsions can be generated in various industries such as aluminum, steel, textiles, leather, food, petrochemicals and metal finishing industries, among others (Wen et al. 2010).

A stable emulsion does not allow the breakage of the phases within a reasonable period of time, and may take years to undo. Some important aspects influence the formation of such emulsions, such as the type of emulsifier, time and stirring intensity, and temperature (Magdich 1988).

Increasing the water content in the W/O type emulsions is also a method used to facilitate the destabilization of the emulsions. However, when a certain volume of water is reached, it will no longer be incorporated into the oil, since saturation of the system is reached. The interfacial properties also have an important influence on the demulsification process, since the degree of interfacial elasticity is positively correlated with the performance of the demulsifier (Wen et al. 2010).

Demulsification consists of two-step process. Flocculation is the first step, in which droplets aggregate and even touch each other at certain points, forming flocs. Next, coalescence occurs, in which water droplets coalesce to form larger droplets. The reduction in the overall quantity of water droplets eventually leads to demulsification (Kokal 2005).

As a type of biosurfactant, a biodesemulsifier is usually efficient in breaking down petroleum emulsions, as well as other industrial emulsions because of their unique functional groups, which cannot be chemically synthesized. Neu (1996) correlated the molar mass of the biosurfactants with their characteristics, concluding that most of the microorganisms produce emulsifiers with high molar mass, while a smaller portion produces compounds with reduced molar mass, which have demulsifying characteristics.

In the demulsification process, the biodemulsifier is adsorbed to the water–oil interface and reacts with the emulsifier, resulting in the removal of the thin film from the surface of the droplets in the emulsion, which causes coalescence, followed by the settling of the droplets and clarification of the continuous phase (Liu et al. 2011a, b).

According to Uzoigwe et al. (2015), it is important to emphasize that the ability to reduce surface and interfacial tensions is considered as a way to differentiate so-called biosurfactants from bioemulsifiers. However, it is not yet clear why bioemulsifiers do not show significant variations in surface and interfacial tension between the different phases (solid–liquid, liquid–liquid and liquid–air).

Bioemulsifiers typically have a higher molecular weight than biosurfactants, since they normally come from complex mixtures of proteins, lipoproteins, lipopolysaccharides among others components (Sekhon-Randhawa 2014). However, surfactants have lower molecular weight and act directly to reduce surface tension, facilitating the destabilization of emulsions Willumsen and Karlson (1997). Rahman et al. (2002), Develter and Lauryssen (2010) and Joshi-Navare et al. (2013) all reported *P. aeruginosa*, *C. bombicola* and *C. tropicalis* have low molecular weight biosurfactants, thus possessing demulsifying characteristics.

Various microorganisms may be used to modify the properties of an emulsion, using hydrophobic cell surfaces or the amphipathic nature of biosurfactants, to displace or modify emulsifiers present at the oil–water interface (Das 2001). The bacteria belonging to the genus *Nocardia*, *Corynebacterium*, *Rhodococcus*, *Mycobacterium* and *Bacillus*, are examples of microorganisms producing biosurfactants, with demulsification activity (Liu et al. 2011a).

Compared with conventional chemical demulsifiers, bioemulsifiers have lower toxicity, environmental compatibility and high efficiency under extreme conditions (Huang et al. 2012; Liu et al. 2010). However, studies on biodemulsifiers still remain at a preliminary stage. Most studies focus only on the selection of bacteria producing biodemulsifiers and on the evaluation of demulsification performance. Kerosene-water type emulsions are generally used in these studies, whereas studies with crude oil emulsions are rarely seen so that the results cannot be extrapolated as to the performance of the biodemulsifiers in the demulsification of petroleum emulsions. In addition, the yield of the production of biodemulsifiers is a crucial issue to allow its industrial application.

In this work, seven biosurfactants, four of which were produced by yeasts of the genus *Candida* and three produced by bacteria of the genera *Pseudomonas* and *Bacillus*, were tested as potential demulsifiers of motor oil emulsions in distilled water and sea water, as well as in model emulsions, to establish potential applications in dispersion of emulsions produced in marine oil spills.

## Materials and methods

### Materials

All reagents used are of analytical grade. The lubricant motor oil (waste oil of car engine or simply motor oil) was obtained from a local automotive workshop and used as the petroleum derivate contaminant. Motor oil was used as contaminant oil that is commercially available for use in flex engines (gasoline, VNG and alcohol), type SAE 20 W-50, with synthetic guard (PETROBRAS). It consists of a paraffinic base lubricating oil (a complex mixture of hydrocarbons) and performance enhancing additives.

### Microorganisms

*Candida lipolytica* UCP0988*, Candida sphaerica* UCP0995*, Candida guilliermondii* UCP0992, *Pseudomonas cepacia* CCT6659*, Pseudomonas aeruginosa* UCP0992 and *Bacillus* sp. were used in the production of biosurfactants. The yeasts and the bacterium *P. aeruginosa* were obtained from the culture collection of the Catholic University of Pernambuco, Recife, state of Pernambuco, Brazil, while *P. cepacia* CCT6659 was obtained from the culture collection of the André Tosello Research and Technology Foundation in the city of Campinas, state of São Paulo, Brazil.

### Biosurfactants

The biosurfactants examined for the demulsification process were produced and characterized for surface tension, critical micelle concentration (CMC) and structure from purified extracts following the procedures described previously, as listed in Table 1. As some of the biosurfactants tested in this work are still being characterized regarding structure, their structures are identified as “probably”.

**Table 1:** Production media, cultivation conditions, CMC, yields, structures and references of the biodemulsifiers tested

Microorganisms	Production medium	Cultivation condition	CMC (mg/l)	Biosurfactant structure	Biosurfactant yield (g/l)	Reference
*C. sphaerica*	Distilled water supplemented with 9% soybean oil refinery residue + 9% corn steep liquor	28 °C and 200 rpm for 144 h	250	Glycolipid	9.0	Luna et al. (2013)
*C. lipolytica (a)*	Mineral medium supplemented with 6% soybean oil refinery residue + 1% glutamic acid	28 °C and 150 rpm for 72 h	300	Lipopeptide	8.0	Rufino et al. (2014)
*C. lipolytica (b)*	Distilled water supplemented with 5% animal fat + 2.5% corn steep liquor	28 °C and 200 rpm for 144 h	800	Glycolipid	2.2	Santos et al. (2013, 2017)
*C. guilliermondii*	Distilled water supplemented with 2.5% molasses + 4.0% corn steep liquor + 2.5% soybean oil refinery residue	28 °C and 200 rpm for 144 h	4200	Probably a glycolipid	2.1	Sarubbo et al. (2016)
*P. cepacia*	Mineral medium supplemented with 2% waste frying oil + 3% corn steep liquor	30 °C and 200 rpm for 144 h	156	Probably a glycolipid	5.2	Rocha e Silva et al. (2014)
*Bacillus* sp.	Mineral medium supplemented with 3% sugar cane molasses + 3% corn steep liquor	27 °C and 200 rpm for 120 h	5000	Probably a lipopeptide	10.5	Chaprão et al. (2015)
*P. aeruginosa*	Mineral medium supplemented with 3% glycerol + 0.6% sodium nitrate	28 °C and 200 rpm for 96 h	700	Glycolipid	8.0	Silva et al. (2010)

### Production of emulsions

To determine the demulsification ability of biosurfactants, emulsions were first prepared by mixing motor oil and distilled water or motor oil and sea water in the ratio 1:1 (v/v) with a digital mechanical mixer at 900 rpm for 15 min. The emulsions were identified as oil-in-water (O/W) type. The prepared emulsion was allowed to stand for 24 h at 28 °C. The fresh emulsions showed less than 5% of emulsion breaking ratio within 24 h. Emulsions of motor oil and distilled water or motor oil and sea water were also treated with SDS (Sodium Dodecyl Sulfate) as control.

### Evaluation of demulsification performance

In the demulsification test, 2 ml of the crude biosurfactants (cell-free broth) or certain concentrations of the isolated biosurfactants (at ½ CMC, the full CMC and twice the CMC) or the chemical surfactant was added to a 20 ml graduated test tube containing 18 ml of the motor oil/distilled water or motor oil/sea water emulsion. The test tubes were vigorously inverted 200 times to achieve complete mixing and then left undisturbed at 28 °C (Liu et al. 2011a; Huang et al. 2009).

Demulsification performance on motor oil emulsions and model emulsions were evaluated by determining percentage oil separation ratio, water separation ratio and emulsion breaking ratio using the following equations:

1 $$oil\;separation\;ratio = \frac{volume\;of\;separated\;top\;oil\;layer}{volume\;of\;oil\;in\;the\;original\;emulsion} \times 100$$

2 $$water\,separation\,ratio = \frac{volume\,of \,water\, on\, the\, botom}{volume\, of \,water\, in\, the\, original \,emulsion + volume \,of \,added}$$

3 $$demulsification \, ratio = \frac{1 - volume \, of \, remaining \, emulsion}{volume \, of \, original \, emulsion + volume \, of \, added \, sample} \times 100$$

### Preparation of oil-in-water (O/W) and water-in-oil (W/O) model emulsions

Oil-in-water (O/W) and water-in-oil (W/O) model emulsions were prepared according to Nadarajah et al. (2002) and Huang et al. (2009), respectively. Stock solution of kerosene was prepared by mixing 0.8 g of Span 80 with 1 l of kerosene on a stir plate. Stock solution of Tween 80-water was prepared by dissolving 1 g of Tween 80 in 1 l of de-ionized water. The solutions were stored in a glass bottle and stirred for 1 min before each use. Emulsions were prepared by adding aqueous and organic (kerosene) components containing the emulsifiers to a 10-ml test tube and mixing them with a vortex at maximum speed until no further emulsification occurred (approx. 5 min). The phase volume ratio of 2:3 was chosen as model emulsion for the demulsification studies using the biosurfactants.

To prepare O/W model emulsions, 200 ml of kerosene containing 0.8 g/l of Span 80 and 300 ml of distilled water containing 1 g/l of Tween 80 were mixed, while the W/O model emulsion was prepared by mixing 300 ml kerosene containing 1 g/l Span 80 and 19 g/l Tween 80 and 200 ml distilled water.

### Surface and interfacial tension determination

The surface tension was measured using a Sigma 700 digital surface tensiometer (KSV Instruments LTD—Finland) working on the principle of the Du Nuoy ring method (Chaprão et al. 2015).

The O/W interfacial tension was measured at ambient temperature according to the procedure described by Wen et al. (2010). The oil phase was produced by dissolving the emulsifiers (2%, percentage by mass) into the kerosene. The Span 80 and Tween 80 mass ratio was 19:1, which corresponds to that used in preparation of W/O model emulsion. The water phase was the cell-free broth after fermentation (crude biosurfactant).

### Isolation of the biosurfactants

The seven biosurfactants tested were isolated according to previous literature. Briefly, the biosurfactants from *C. sphaerica* and *C. guilliermondii* were isolated according to Pareilleux (1979) The biosurfactant from *C. lipolytica* (a) was isolated as described by Cirigliano and Carman (1984), while the biosurfactant from *C. lipolytica* (b) was isolated according to Ilori et al. (2005). The bacterial biosurfactants were all isolated as described by Costa et al. (2006).

### Critical micelle concentration (CMC)

The critical micelle concentration (CMC) was determined by measuring the surface tensions of dilutions of isolated biosurfactant in distilled water up to a constant value of surface tension. Stabilization was allowed to occur until standard deviation of 10 successive measurements was less than 0.4 mN/m. Each result was the average of 10 determinations after stabilization. The value of CMC was obtained from the plot of surface tension against surfactant concentration. The CMC value was determined to be g/l of biosurfactant.

### Cell surface hydrophobicity

Cell surface hydrophobicity (CSH) was measured by cells adherence to hydrocarbons (MATH), as described in Coimbra et al. (2009). Cells were washed twice and resuspended in a buffered salt solution (16.9 g/l K_2_HPO_4_, 7.3 g/l KH_2_PO_4_) to give an OD at 600 nm of 0.5. The cell suspension (2.0 ml) with 100 µl kerosene added was vortex shaken for 3 min in glass tubes (10 × 100 mm). After shaking, kerosene and aqueous phases were allowed to separate for 1 h. The OD of the aqueous phase was then measured at 600 nm. Hydrophobicity was expressed as the percentage of cell adherence to kerosene calculated as follows:

4 $$hydrophobicity = 1 - \frac{OD \, of \, aqueous \, phase}{OD \, of \, initial \, cell \,suspension} \times 100$$

For a given sample, three independent determination were carried out. High hydrophobicity values indicate high affinity of the cells for oils.

### Statistical analysis

In order to verify the existence of differences between the average responses of the treatments, when having more than two groups, it is inappropriate to simply compare each pair using a t test because of the problem of multiple testing. In this case, it was used a one-way analysis of variance (ANOVA) to evaluate whether there was any evidence that the means of the populations differed (Kim 2017). Since the ANOVA leaded to a conclusion that there was evidence that the group means differ, it was investigated whether which of the means were different. In this case, Tukey’s honestly significant difference test (Tukey’s HSD) was used. This test compared the difference between each pair of means with appropriate adjustment for the multiple testing.

## Results

### Demulsification performance of the biosurfactants

Seven biosurfactants were tested in order to analyze their demulsification capacity (Table 1). All of them were produced in low-cost substrates. Table 2 presents the demulsifying performance of the seven crude biosurfactant extracts and isolated biosurfactants tested on motor oil emulsions. The crude biosurfactant extracts is the cell-free fermentation broth obtained while the isolated biosurfactant is the biomolecule obtained after extraction with solvent.

**Table 2:** Demulsification percentage of motor oil emulsions in distilled water and in sea water after addition of biosurfactants

Microorganisms	Biosurfactant concentration	Demulsification of motor oil (%)^a^	
		Oil-in-water (O/W) emulsions	
		Distilled water	Sea water
*C. sphaerica*	Cell-free broth	39.0 ± 0.9	40.6 ± 0.5
	½ CMC	35.0 ± 0.9	38.2 ± 0.6
	CMC	36.9 ± 0.7	38.4 ± 0.7
	2 × CMC	37.0 ± 0.9	39.4 ± 0.8
*C. lipolytica (a)*	Cell-free broth	30.0 ± 0.9	36.0 ± 0.9
	½ CMC	37.9 ± 0.4	37.0 ± 0.9
	CMC	37.0 ± 0.9	42.4 ± 0.8
	2 × CMC	35.7 ± 0.3	43.0 ± 0.9
*C. lipolytica (b)*	Cell-free broth	27.3 ± 0.7	26.9 ± 0.9
	½ CMC	27.0 ± 0.6	27.0 ± 0.9
	CMC	27.0 ± 0.9	26.7 ± 0.6
	2 × CMC	27.0 ± 0.5	27.3 ± 0.5
*C. guilliermondii*	Cell-free broth	31.7 ± 0.9	44.9 ± 0.9
	½ CMC	40.0 ± 0.7	40.7 ± 0.4
	CMC	42.6 ± 0.3	44.6 ± 0.9
	2 × CMC	42.1 ± 0.9	41.0 ± 0.9
*P. cepacia*	Cell-free broth	30.6 ± 0.7	39.0 ± 0.9
	½ CMC	34.0 ± 0.9	45.0 ± 0.9
	CMC	43.3 ± 0.7	37.5 ± 0.5
	2 × CMC	43.8 ± 0.5	65.0 ± 0.9
*Bacillus* sp.	Cell-free broth	42.0 ± 0.9	37.0 ± 0.9
	½ CMC	46.6 ± 0.9	40.0 ± 0.8
	CMC	47.0 ± 0.9	54.4 ± 0.9
	2 × CMC	60.1 ± 0.8	66.0 ± 0.9
*P. aeruginosa*	Cell-free broth	44.0 ± 0.5	50.0 ± 0.6
	½ CMC	21.2 ± 0.4	29.1 ± 0.8
	CMC	48.0 ± 0.9	50.0 ± 0.9
	2 × CMC	62.0 ± 0.9	65.7 ± 0.6

Results are expressed as mean ± pure error

^a^Control values for the demulsification in the absence of biosurfactants were less than 5%

The values of the demulsification rates indicate the destabilizing value of the emulsions, that is, the percentage of water recovered. Thus, it is observed that, regardless of the type of water used (distilled or sea water), in all types of biosurfactants tested and in all the concentrations used, demulsification of motor oil occurred, with percentages varying between 26 and 66% demulsification. The chemical surfactant SDS used as control was able to separate 80% of the motor oil in both distilled and sea water.

The application of statistical tests (Kim 2017) indicated the best de-emulsification performances for the different biosurfactants applied in distilled water and seawater, according to Table 3. This table summarizes Table 2, indicating the respective treatments with the best performances.

**Table 3:** Indication of the biosurfactants that showed the best demulsification results identified by the statistical tests

Biosurfactant concentration	Demulsification of motor oil (%) by the biosurfactants produced	
	Distilled water	Sea water
Cell-free broth	*P. aeruginosa* (44.0 ± 0.5)	*P. aeruginosa* (50.0 ± 0.6)
½ CMC	*Bacillus* sp. (46.6 ± 0.9)	*P. cepacia* (45.0 ± 0.9)
CMC	*P. aeruginosa* (48.0 ± 0.9)	*Bacillus* sp. (54.4 ± 0.9)
2 × CMC	*P. aeruginosa* (62.0 ± 0.9)	*Bacillus* sp. (66.0 ± 0.9)

The biosurfactant from *C. sphaerica* presented superior demulsification results for seawater tests, the best result being with the use of the cell-free broth. However, it is noteworthy that the metabolic broth presented a significant result also in distilled water, since the demulsification rate in this condition presented values statistically equal to the other tests in seawater, when compared to the concentrations of the isolated surfactant (½ CMC, CMC and 2 × CMC).

The biosurfactant from *C. lipolytica* (a) also presented better results for seawater tests. However, the best results were obtained for the concentrations of the isolated biosurfactant in the CMC and twice the CMC, both statistically equal. The other concentrations presented similar values of demulsification in both seawater and distilled water.

The biosurfactant from *C. guilliermondii* also presented superior results for the tests performed with seawater. The tests with the isolated biodemulsifier in the middle of the CMC and in the CMC demonstrated statistical equality, showing that it is more advantageous to use the lower concentration, considering the economic and toxicological factor. On the other hand, the statistical treatment showed that the results obtained for the biosurfactant from *C. lipolytica* (b) were statistically similar, regardless of the biosurfactant concentration or type of water used, presenting mean values of 27% demulsification.

Briefly, in most seawater tests, the percentages were higher than those observed in distilled water. In other words, the best separations of water from motor oil were achieved for the samples containing NaCl compared to the oil in distilled water. This phenomenon can be explained by the change in interfacial film behavior. The salt ions leading to an increase in relaxation of the formed film, as described by Binks (1993).

The best results were observed for the biosurfactants produced by bacteria at twice the CMC concentration, which demulsified around 65% of the water, which is statistically significant higher than those obtained in tests performed with yeast species. Thus, it can be observed that the biosurfactants produced by the bacteria showed greater effectiveness than those originating from yeasts for the demulsification activities.

It was possible to observe, in general, that the isolated biosurfactants presented demulsification capacity superior to the cell-free broth (crude biosurfactants). In the specific case of yeast biosurfactants, the increasing of the concentration of the isolated surfactants (at ½ CMC, CMC and twice the CMC) did not increase the demulsification ratio, showing that the lower concentration would already be enough to de-emulsify at a lower cost.

**Fig. 1:**
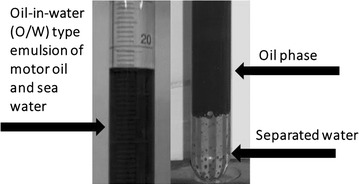
Desestablization of motor oil emulsion by the biosurfactant from *Bacillus* sp.

Figure 1 illustrates the separation of the aqueous phase from the motor oil emulsion after addition of the biosurfactant from *Bacillus* sp.

### Demulsification of W/O and O/W model emulsions

Model kerosene–water emulsions were also used to explore the potential of the biosurfactants to demulsify oil emulsions. Therefore, water-in-kerosene model emulsions stabilized with Span-80 and Tween-80 surfactants was developed. To prepare oil-in-water and water-in-oil model emulsions kerosene has been used as the organic phase. Although some spontaneous separation was observed in the untreated control tubes, the treated tubes showed a clear separation of phases, namely the top kerosene phase, mid interface and the bottom aqueous phase.

Samples of the cell-free broth (crude biosurfactants) were evaluated for their ability to break W/O (Tween–kerosene) and O/W (Span–Tween–kerosene) emulsions. The results are described in Table 4.

**Table 4:** Demulsification performance on W/O (Tween–kerosene) and O/W (Span–Tween–kerosene) model emulsions by the crude biosurfactants (cell-free broth)

Biosurfactant producers	Model emulsions demulsification (%)^a^	
	W/O	O/W
*C. sphaerica*	90.0 ± 0.9	94.7 ± 0.4
*C. lipolytica (a)*	38.2 ± 0.9	35.7 ± 0.4
*C. lipolytica (b)*	44.0 ± 0.9	45.2 ± 0.9
*C. guilliermondii*	30.0 ± 0.9	32.0 ± 0.9
*P. cepacia*	30.0 ± 0.9	33.4 ± 0.8
*Bacillus* sp.	37.0 ± 0.9	41.0 ± 0.9
*P. aeruginosa*	35.0 ± 0.9	38.2 ± 0.5

Results are expressed as mean ± pure error

^a^Control values for the demulsification in the absence of biosurfactants were less than 5%

Statistical tests (Kim 2017) were also applied in the analysis of the data in Table 4. The biosurfactant from *C. sphaerica* presented significant results for both W/O and O/W model emulsions, with demulsification values of 90 ± 0.9 and 94.7 ± 0.4, respectively. On the other hand, the other biosurfactants presented a statistically similar behavior, with no great variation or significance of the demulsification percentages. However, it is still possible to observe that the biosurfactants had a higher demulsification rate for O/W type emulsions, except for the biosurfactant from *C. lipolytica* (b), which showed demulsification values around 45%.

A clear separation of phases was demonstrated in tubes containing W/O emulsion, kerosene–Tween–Span emulsion and biosurfactants, with an aqueous phase at the bottom and kerosene phase at the top and interface. Different results were found regarding the motor oil emulsion, with a film of oil at the top of the tube and a turbid phase throughout the rest of the emulsion. The use of Tween 80 (hydrophilic surfactant) or Span 80 (hydrophobic surfactant) had an immediate effect on the destabilization of the emulsion, with maximum demulsification achieved at 24 h.

**Fig. 2:**
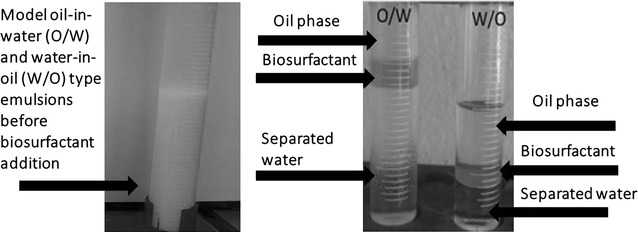
Phases separation in W/O (Tween–kerosene) and O/W (Span–Tween–kerosene) model emulsions after addition of the biosurfactant from *C. sphaerica*

Figure 2 illustrates the emulsions destabilized and undone after the addition of the biosurfactants, showing separation of the phases.

### Relationship between interfacial tension, cell hydrophobicity and demulsification

The physiochemical properties such as cell surface hydrophobicity, surface and interfacial activity can influence the demulsification ability of demulsifying strains (Liu et al. 2011a; Raza et al. 2006). Thus, the surface tension and interfacial tension and the cell hydrophobicity of the biosurfactants were measured and are shown in Table 5.

**Table 5:** Cell hydrophobicity, surface tension, and interfacial tension values obtained after cultivation of bacteria and yeast species in their respective medium for biosurfactant production

Microorganisms	Cellular hydrophobicity (%)	Surface tension (mN/m)	Interfacial tension (mN/m)
*C. sphaerica*	64.2 ± 0.5	25.0 ± 0.7	12.4 ± 0.2
*C. lipolytica (a)*	79.1 ± 0.6	25.0 ± 0.9	12.6 ± 0.5
*C. lipolytica (b)*	85.5 ± 0.6	27.7 ± 0.5	13.4 ± 0.6
*C. guilliermondii*	50.5 ± 0.3	31.0 ± 0.6	15.3 ± 0.8
*P. cepacia*	80.3 ± 0.7	25.5 ± 0.9	13.7 ± 0.5
*Bacillus* sp.	73.2 ± 0.5	29.0 ± 0.7	14.5 ± 0.3
*P. aeruginosa*	82.3 ± 0.4	26.0 ± 1.0	12.8 ± 0.4

Results are expressed as mean ± pure error

Regarding cell hydrophobicity, most microorganisms presented values above 70%, except for *C. sphaerica* and *C. guilliermondii*, which presented values of 64 and 50%, respectively, as evaluated by MATH. The greater the hydrophobicity of the cell surface, the greater the chances of success in the adhesion of the oil particles and subsequent destabilization of the emulsions. However, it was not possible to describe a correlation between cellular hydrophobicity and demulsification rates for model emulsion tests, since the results demonstrate an opposite-to-expected behavior for some biosurfactants. This may be due to the fact that cell hydrophobicity was obtained using the washed cells while demulsification tests were carried out on cell-free products of these strains. The biosurfactant from *P. aeruginosa*, for example, which showed the second highest hydrophobicity (82%), showed the lowest motor oil demulsification at ½ CMC in seawater and distilled water (Table 2). Regarding the biodemulsifiers from yeasts, two exceptions were also found, i.e., the cell surface hydrophobicity of *C. lipolytica* (b) cultivated in medium containing animal fat and corn steep liquor was high, although the demulsification capacity did not exceed 28%. The same was observed for the biosurfactant from *C. sphaerica*. As it was not possible to find a correlation for these behaviours, neither analysing the CMC values obtained for these biodemulsifiers, we believe that these values are a consequence of the steric conformation of these biomolecules within the emulsions.

It is known that interfacial tension is decreased due to the adsorption of surfactants at the interface of liquids with the polar end in water and the hydrocarbon chain in the oil. Thus, the lower the interfacial tension, the better the oil mobility and, consequently, the better its efficiency in the destabilization process of oily emulsions (Santos et al. 2016). All biosurfactants presented similar interfacial tensions from a statistical point of view, with a slight highlight for the biosurfactants from *C. sphaerica*, *C. lipolytica* (a) and *P. aeruginosa*, which presented values below 13 mN/m. Since the demulsification percentages varied between the biosurfactants, the interfacial tension values do not appear to contribute to the demulsifying capability and therefore may not be a key factor for the ability of the biosurfactants to act as demulsifiers.

As for the surface tension, the biosurfactants from *C. sphaerica*, *C. lipolytica* (a), *P. cepacia* and *P. aeruginosa* presented statistically similar values, between 25 and 26 mN/m, which can be considered excellent when compared with surface tension values described in literature. However, although the surfactants produced by *Bacillus* sp. and *C. guilliermondii* presented surface tension values around 30 mN/m, they can still be considered as good surfactants in comparison with other studies (Santos et al. 2016).

### Microscopic observations

**Fig. 3:**
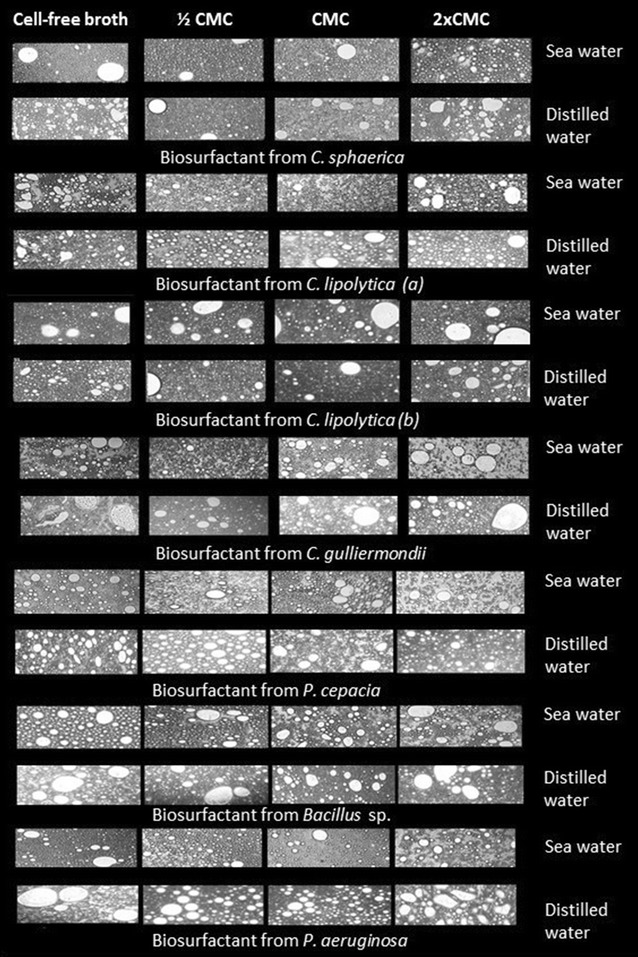
Microscopy (×40 magnification) of the residual motor oil emulsions in sea water and distilled water with the crude (cell-free broth) and the isolate biosurfactants (at ½ CMC, at the CMC and 2 × CMC) at 24 h after initiation of the demulsification process

To study the changes of emulsion droplets, microscopy was used to observe the emulsion during demulsification process. The microscopy of the residual motor oil emulsions in distilled water and in sea water with the crude and isolated biosurfactants after 24 h initiation of the demulsification process is shown in Fig. 3.

It can be observed that, in general, the higher the concentration of the biosurfactant in the emulsion, the greater the amount and size of the droplets, facilitating the demulsification. In the case of the crude biosurfactants, i.e., the cell-free broth, a particular behavior was observed for each biosurfactant tested, since these preparations have other impurities and metabolites in their composition. It is also possible to observe that the mixtures containing the bacterial biosurfactants (*Bacillus* and *Pseudomonas*) have uniform aspect with regard to the size of the droplets, corroborating the superior demulsification results for these biosurfactants, as shown in Table 2.

Studies have shown that the size of droplets, as well as the shape of droplet size distribution, depends on several factors, including interfacial tension, shear rate (mixtures, accidents, etc.), the nature of the emulsifier, the presence of solids, and the properties of the oil and the aqueous phase. Generally, it is considered that the smaller the droplet size of the dispersed phase, the more stable the emulsion is (Kokal 2005). Thus, it is possible to observe that the smaller droplet size is visualized in the presence of lower concentrations of biosurfactants, which demonstrates the lower percentage of demulsification observed in Table 2.

Figure 3 also shows that the residual motor oil emulsion droplets, obtained after the action of the biodemulsifiers, are smaller in the presence of sea water, corroborating the results obtained in Table 2, which shows that the demulsification percentages were higher when the contaminant was dispersed in sea water. In other words, the presence of salt reduces the stability of O/W emulsions.

Regarding the size of droplets observed in the microscope for the motor oil emulsions, it was possible to verify that the residual oil phases in sea water were more compact after addition of the demulsifiers, i.e., the presence of NaCl facilitated the separation of water.

## Discussion

The chemical treatment of water–oil emulsions by the addition of appropriate demulsifiers is widely used in the breaking/destabilization of such emulsions and consequently in the separation of the oil and water phases. The demulsifiers present interfacial properties and adsorb at the water–oil interface, changing their physical–chemical properties and thus favoring the coalescence between water droplets (Kokal 2005).

All biosurfactants tested in this work showed demulsification capacity. It is important to highlight that the results obtained can be considered satisfactory, since most of the biosurfactants tested here were produced from industrial residues, making them more attractive since they can be used in the crude form (Santos et al. 2016). It is also worth mentioning that the studies found in the literature do not describe the use of biosurfactants produced by yeasts as demulsifying agents. It seems that these are the first results published with these agents from yeasts.

From the results obtained, which showed that there were no large differences in the percentage of demulsification between the crude extracts and the isolated biosurfactants, some considerations can be made. In an industrial application of demulsifiers, the proper balance between the cost of using a higher concentration to save time and equipment capacity and a lower concentration and cost of demulsifier, which will result in longer separation time and probably a higher investment in the capacity of equipment should be evaluated, as discussed by Hajivand and Vaziri (2015) who found that the lowest concentration of fatty alcohol ethoxylate gave 52% separation, while the highest concentration of the demulsifier achieved 64% separation.

The results obtained here also demonstrated that the ability to use the crude or isolated biosurfactant will depend on the type of biomolecule, since the percentages varied between the biosurfactant states. Liu et al. (2010) applied the biodemulsifier from *Alcaligenes* sp. S-XJ-1 grown in paraffin. The 10% (v/v) fermented broth and 120 mg/l powder-dried biodemulsifier resulted in demulsification rates of 98 and 95%, respectively, when applied to a crude oil emulsion, demonstrating the somewhat lower degree of effectiveness of the isolated biodemulsifier in comparison to the crude broth. Li et al. (2012), on the other hand, showed that the combination of glucose and liquid paraffin as carbon sources increased 35.5% of the demulsifying ratio of the biodemulsifier produced by *B. mojavensis* XH1.

Other works also describe the application of biodemulsifiers produced from industrial wastes, as the ones tested in this work. *Dietzia* sp. S-JS-1 was used to synthesize a demulsifier using waste frying oils as substrate; after 5 h, the demulsifier separated 88.3% of the oil from a W/O emulsion and 76.4% of the water from a O/W emulsion (Liu et al. 2009). Another demulsifying strain of *Alcaligenes* sp. S-XJ-1 could also use waste frying oil as carbon source. The emulsion separation ratio of the biodemulsifier after 24 h was 47% (Liu et al. 2011b). At a concentration of 356 mg/l, substances produced by *Alcaligenes* sp. S-XJ-1 achieved a 67.5% separation ratio of water-in-kerosene emulsions (Huang et al. 2013). The bacterium *Paenibacillus alvei* ARN63 was used to produce a demulsifier to break down a water-in-heavy crude oil emulsion; the best carbon source was motor oil and the demulsification ratio reached nearly 77% (Amirabadi et al. 2013).

The biosurfactants tested in this work were also able to act as demulsifiers of model emulsions. Coutinho et al. (2013) found that the cells and metabolites produced by *P. aeruginosa* have demulsifying characteristics for W/O and O/W emulsions, with a demulsification rate higher than 78%, showing the potential of the bacteria to be applied in the treatment of effluents and in industrial applications such as oil processing and tank cleaning. Studies conducted by Wen et al. (2010) using biomass from some microorganisms showed that the increase in the number of cells in the emulsions also increased the demulsification activities. Another way of improving the efficiency of emulsion destabilization is described by Long et al. (2013), who found that the increase in pH positively influenced the demulsification of W/O emulsions, a result similar to that obtained in crude oil emulsions. In contrast, pH reduction showed positive results for O/W type emulsions. The rhamnolipid showed over 90% of demulsification efficiency on refractory waste crude oil which was confirmed on model emulsions.

The cell surface properties of demulsifying bacteria cells can be controlled by cell surface substances. Bacterial cell surface hydrophobicity is one of the most significant features that determines bacterial adhesion to an oil–water interface, which can accelerate cell transfer to the water–oil interface due to an improved affinity with oil and strengthen the aggregation of dispersed droplets in the W/O emulsion (Liu et al. 2011a, b). The results obtained in this work showed that it was not possible to describe a correlation between cellular hydrophobicity values obtained for the washed microbial cells used to produce the tested biosurfactants and the demulsification rates obtained using the cell-free biosurfactants produced by these microbial cells.

The amplitude of interfacial waves is determined by interfacial tension. A reduction in interfacial tension leads to an increase in the amplitude of the waves. This causes adjacent droplets to approach a critical distance and coalesce, resulting in demulsification (Sjöblom et al. 1992). In the conceptual model of such coalescence, two water droplets approach one another due to the thinning of the film following the outflow of liquid. In this process, the adsorbed surfactant is carried away and a surfactant concentration gradient is created. As a result, an interfacial tension gradient is established to counteract the thinning and ensure the stability of the emulsion (Kocherginsky et al. 2003).

Chemical demulsifiers with higher interfacial tension have been shown to enhance breakup of the interfacial film and to increase the coalescence of droplets (Kang et al. 2006; Kim and Wasan 1996; Krawczyk et al. 1991; Deng et al. 2005). Our results are in accordance with Fernandes et al. (2014) who stated that the ability to break W/O emulsions by bacterial isolates is not always related to CSH and to production of biosurfactants that exhibit demulsification activity.

The size of droplets for the motor oil emulsions was also evaluated after addition of the demulsifiers in distilled and sea water. According to Moradi et al. (2011) who studied the impact of salinity on crude oil/water emulsions by measuring the droplet-size distribution visualized by an optical microscopy method, emulsions are more stable at lower ionic strength of the aqueous phase. According to Binks (1993), the presence of salt seems to have an adverse effect on emulsion stability.

Our results highlight the potentials for an ability to produce surface active materials by selected microorganisms that can be employed for demulsification activities, and how some may be better producers than other for such uses, we hope that this leads to further investigations in this area. The data certainly provides preliminary indications rather than quantitative analysis. According to Kokal (2005), the droplet size distribution influences the viscosity of the emulsion, that is, the emulsions are more viscous when the droplets are of smaller size and also when the distribution is more compact and with more uniform droplet sizes. Thus, increased stability can be attributed to the high viscosities found in emulsions with small droplet sizes, making the demulsification process difficult.

Considering that this is the first study involving these biosurfactants as biodemulsifiers, the results can be considered satisfactory since high demulsification percentages can be reached depending on the conditions of application and that the fresh emulsions showed less than 5% of emulsion breaking ratio within 24 h, as described in “Materials and methods” section. As oil producers are interested in the speed and in the amount of water left after separation, these biomolecules can be promising demulsification agents in the future according to the exigencies of a petroleum industry.

The biosurfactants tested showed abilities to act as demulsifying agents when used isolated or in their crude form. The possibility of using crude preparations of the biosurfactants, in particular, can favor the application of these agents on a large scale. In this way, it is possible to verify the environmental application of these biotechnological agents as an adjunct to the processes of recovery of oil spilled to the refineries and to the treatment of sea water, collaborating not only for the conservation of the environment, but also for the reduction of the costs of the petrochemical industries with maritime accidents. The use of environmentally friendly demulsifiers for the breakdown of hydrocarbon-water emulsions encountered in crude oil production is also a very important tool to allow petroleum industries to recover a product of improved quality, especially in platforms and may have other environmental and oil–water emulsion waste or contaminations application.

## References

[CR1] Almeida DG, Soares Da Silva RCF, Rufino RD, Luna JM, Santos VA, Banat IM, Sarubbo LA (2016). Biosurfactants: promising molecules for petroleum biotechnology advances. Front Microbiol.

[CR2] Amirabadi SSh, Jahanmiri A, Rahimpour MR, Rafienia B, Darvishi P, Niazi A (2013). Investigation of *Paenibacillus alvei* ARN63 ability for biodemulsifier production: medium optimization to break heavy crude oil emulsion. Colloids Surf B Biointerfaces.

[CR3] Binks BP (1993). Surfactant monolayers at the oil–water interface. Chem Ind.

[CR4] Cambiella A, Ortea E, Ríos G, Benito JM, Pazos C, Coca J (2006). Treatment of oil-in-water emulsions: performance of a sawdust bed filter. J Hazard Mater.

[CR5] Chaprão MJ, Ferreira INS, Correa PF, Rufino RD, Luna JM, Silva EJ, Sarubbo LA (2015). Application of bacterial and yeast biosurfactants for enhanced removal and biodegradation of motor oil from contaminated sand. Electron J Biotechnol.

[CR6] Cirigliano MC, Carman GM (1984). Isolation of a bioemulsifier from *Candida lipolytica*. Appl Environ Microb.

[CR7] Coimbra CD, Rufino RD, Luna JM, Sarubbo LA (2009). Studies of the cell surface properties of Candida species and relation to the production of biosurfactants for environmental applications. Curr Microbiol.

[CR8] Costa SGVAO, Nitschke M, Haddad R, Eberlin MN, Contiero J (2006). Production of *Pseudomonas aeruginosa* LBI rhamnolipids following growth on Brazilian native oils. Process Biochem.

[CR9] Coutinho JOPA, Silva MPS, Moraes PM, Monteiro AS, Barcelos JCC, Siqueira EP, Santos LL (2013). Demulsifying properties of extracellular products and cells of *Pseudomonas aeruginosa* MSJ isolated from petroleum-contaminated soil. Bioresour Technol.

[CR10] Das M (2001). Characterization of de-emulsification capabilities of a *Micrococcus* sp. Bioresour Technol.

[CR11] Deng SB, Yu G, Jiang ZP, Zhang RQ, Ting YP (2005). Destabilization of oil droplets in produced water from ASP flooding. Colloids Surf A.

[CR12] Develter DWG, Lauryssen LML (2010). Properties and industrial applications of sophorolipids. Eur J Lipid Sci Technol.

[CR13] Feng X, Mussone P, Gao S, Wang S, Wu SY, Masliyah JH, Xu Z (2009). Mechanistic study on demulsification of water-in-diluted bitumen emulsions by ethylcellulose. Langmuir.

[CR14] Fernandes RCR, Borges AC, Tótola MR (2014). Cellular hydrophobicity is not determinant of water-in-oil emulsification breaking by bacteria. Int J Appl Sci Technol.

[CR15] Hajivand P, Vaziri A (2015). Optimization of demulsifier formulation for separation of water from crude oil emulsions. Braz J Chem Eng.

[CR16] Huang X, Liu J, Lu L, Wen Y, Xu J, Yang D, Zhou Q (2009). Evaluation of screening methods for demulsifying bacteria and characterization of lipopeptide bio-demulsifier produced by *Alcaligenes* sp. Bioresour Technol.

[CR17] Huang XF, Li MX, Lu LJ, Yang S, Liu J (2012). Relationship of cell-wall bound fatty acids and the demulsification efficiency of demulsifying bacteria *Alcaligenes* sp. S-XJ-1 cultured with vegetable oils. Bioresour Technol.

[CR18] Huang X, Peng K, Feng Y, Liu J, Lu L (2013). Separation and characterization of effective demulsifying substances from surface of *Alcaligenes* sp. S-XJ-1 and its application in water-in-kerosene emulsion. Bioresour Technol.

[CR19] Ilori MO, Amobi CJ, Odocha AC (2005). Factors affecting biosurfactant production by oil degrading *Aeromonas* spp. isolated from a tropical environment. Chemosphere.

[CR20] Jiang T, Hirasaki GJ, Miller CA (2010). Characterization of kaolinite potential for interpretation of wettability alteration in diluted Bitumen emulsion separation. Energy Fuels.

[CR21] Joshi-Navare K, Khanvilkar P, Prabhune A (2013). Jatropha oil derived sophorolipids: production and characterization as laundry detergent additive. Biochem Res Int.

[CR22] Kang WL, Jing GL, Zhang HY, Li MY, Wu ZL (2006). Influence of demulsifier on interfacial film between oil and water. Colloids Surf A.

[CR23] Kim TJ (2017). Understanding one-way ANOVA using conceptual figures. Korean J Anesthesiol.

[CR24] Kim YH, Wasan DT (1996). Effect of demulsifier partitioning on the destabilization of water-in-oil emulsions. Ind Eng Chem Res.

[CR25] Kocherginsky NM, Tan CL, Lu WF (2003). Demulsification of water-in-oil emulsions via filtration through a hydrophilic polymer membrane. J Membr Sci.

[CR26] Kokal SL (2005). Crude oil emulsion: a state-of-art review. Soc Pet Eng.

[CR27] Krawczyk MA, Wasan DY, Shetty CS (1991). Chemical demulsification of petroleum emulsions using oil-soluble demulsifiers. Ind Eng Chem Res.

[CR28] Li X, Li A, Liu C, Yang J, Ma F, Hou N, Xu Y, Ren N (2012). Characterization of the extracellular biodemulsifier of *Bacillus mojavensis* XH1 and the enhancement of demulsifying efficiency by optimization of the production medium composition. Process Biochem.

[CR29] Liu J, Huang XF, Lu LJ, Xu JC, Wen Y, Yang DH, Zhou Q (2009). Comparison between waste frying oil and paraffin as carbon source in the production of biodeemulsifier by *Dietzia* sp. S-JS-1. Bioresour Technol.

[CR30] Liu J, Lu LJ, Huang XF, Shang JJ, Li MX, Xu JC, Deng HP (2010). Relationship between surface physicochemical properties and its demulsifying ability of an alkaliphilic strain of *Alcaligenes* sp. S-XJ-1. Process Biochem.

[CR31] Liu J, Huang X-F, Lu L-J, Li M-X, Xu J-C, Deng H-P (2011). Turbiscan Lab^®^ expert analysis of the biological demulsification of a water-in-oil emulsion by two biodemulsifiers. J Hazard Mater.

[CR32] Liu J, Peng K, Huang X, Lu L, Cheng H, Yang D, Zhou Q, Deng H (2011). Application of waste frying oils in the biosynthesis of biodemulsifier by a demulsifying strain *Alcaligenes* sp. S-XJ-1. J Environ Sci.

[CR33] Long X, Zhang G, Shen C, Sun G, Wang R, Yin L, Meng Q (2013). Application of rhamnolipid as a novel biodemulsifier for destabilizing waste crude oil. Bioresour Techonol.

[CR34] Luna JM, Rufino RD, Sarubbo LA, Campos-Takaki GM (2013). Characterization, surface properties and biological activity of a biosurfactant produced from industrial waste by *Candida sphaerica* UCP0995 for application in the petroleum industry. Colloids Surf B Biointerfaces.

[CR35] Magdich P (1988) The removal of oil from oil–water mixtures using selective oil filtration. Master of Science in civil engineering. University of Minnesota USA

[CR36] Moradi M, Alvarado V, Huzurbazar S (2011). Effect of salinity on water-in-crude oil emulsion: evaluation through drop-size distribution proxy. Energy Fuels.

[CR37] Nadarajah N, Singh A, Ward O (2002). Evaluation of mixed bacterial culture for de-emulsification of water-in-petroleum oil emulsions. World J Microbiol Biotechnol.

[CR38] Neu TR (1996). Significance of bacterial surface-active compounds in interaction of bacteria with interfaces. Microbiol Rev.

[CR39] Pareilleux A (1979). Hydrocarbon assimilation by *Candida lipolytica*: formation of a biosurfactant: effects on respiratory activity and growth. Eur J Appl Microbiol Biotechnol.

[CR40] Rahman KSM, Thahira-Rahman J, Mcclean S, Marchant R, Banat IM (2002). Rhamnolipid biosurfactants production by strains of *Pseudomonas aeruginosa* using low cost materials. Biotechnol Prog.

[CR41] Raza ZA, Khan MS, Khalid ZM, Rehman A (2006). Production kinetics and tensioactive characteristics of biosurfactant from a *Pseudomonas aeruginosa* mutant grown on waste frying oils. Biotechnol Lett.

[CR42] Rocha e Silva NMP, Rufino RD, Luna JM, Santos VA, Sarubbo LA (2014). Screening of *Pseudomonas* species for biosurfactant production using low-cost substrates. Biocatal Agric Biotechnol.

[CR43] Rufino RD, Luna JM, Campos-Takaki GM, Sarubbo LA (2014). Characterization and properties of the biosurfactant produced by *Candida lipolytica* UCP 0988. Electron J Biotechnol.

[CR44] Santos DKF, Luna JM, Rufino RD, Santos VA, Salgueiro AA, Sarubbo LA (2013). Synthesis and evaluation of biosurfactant produced by *Candida lipolytica* using animal fat and corn steep liquor. J Pet Sci Eng.

[CR45] Santos DKF, Rufino RD, Luna JM, Santos VA, Sarubbo LA (2016). Biosurfactants: multifunctional biomolecules of the 21st century. Int J Mol Sci.

[CR46] Santos DKF, Meira HM, Rufino RD, Luna JM, Sarubbo LA (2017). Biosurfactant production from *Candida lipolytica* in bioreactor and evaluation of its toxicity for application as a bioremediation agent. Process Biochem.

[CR47] Sarubbo LA, Rocha Júnior RB, Luna JM, Rufino RD, Santos VA, Banat IM (2015). Some aspects of heavy metals contamination remediation and role of biosurfactants. Chem Ecol.

[CR48] Sarubbo LA, Luna JM, Rufino RD, Brasileiro PPF (2016). Production of a low-cost biosurfactant for application in the remediation of sea water contaminated with petroleum derivates. Chem Eng Trans.

[CR49] Sekhon-Randhawa KK (2014) Biosurfactants produced by genetically manipulated microorganisms: challenges and opportunities. In: Kosaric N, Sukan FV (ed) Biosurfactants. CRC Press, Boca Raton, pp 49–67

[CR50] Silva SNRL, Farias CBF, Rufino RD, Luna JM, Sarubbo LA (2010). Glycerol as substrate for the production of biosurfactant by *Pseudomonas aeruginosa* UCP0992. Colloids Surf B Biointerfaces.

[CR51] Silva RCFS, Almeida DG, Luna JM, Rufino RD, Santos VA, Sarubbo LA (2014). Applications of biosurfactants in the petroleum industry and the remediation of oil spills. Int J Mol Sci.

[CR52] Sjöblom J, Li MY, Christy AA, Gu T (1992). Water-in-crude-oil emulsions from the Norwegian continental shelf 7. Interfacial pressure and emulsion stability. Colloids Surf.

[CR53] Staiss F, Bohm R, Kupfer R (1991). Improved demulsifier chemistry: a novel approach in the dehydration of crude oil. SPE Prod Eng.

[CR54] Stephenson WK (1990) Alkoxylated vinyl polymer demulsifiers. US patent 4(968)449

[CR55] Uzoigwe C, Burgess JG, Ennis CJ, Rahman PKSM (2015). Bioemulsifiers are not biosurfactants and require different screening approaches. Front Microbiol.

[CR56] Wen Y, Cheng H, Lu L-J, Liu J, Feng Y, Guan W, Zhou Q, Huang X-F (2010). Analysis of biological demulsification process of water-in-oil emulsion by *Alcaligenes* sp. S-XJ-1. Bioresour Technol.

[CR57] Willumsen PA, Karlson U (1997). Screening of bacteria, isolated from PAH-contaminated soil, for production of biosurfactants and bioemulsifiers. Biodegradation.

[CR58] Xia L, Gong K, Wang S, Li J, Yang D (2010). Microwave-assisted chemical demulsification of water-in-crude-oil emulsions. J Dispers Sci Technol.

[CR59] Yang L, Nakhla G, Bassi A (2005). Electro-kinetic dewatering of oily sludges. J Hazard Mater.

[CR60] Zhang J, Li J, Thring RW, Hu X, Song X (2012). Oil recovery from refinery oily sludge via ultrasound and freeze/thaw. J Hazard Mater.

